# Fibroblast growth factor 8 is expressed at higher levels in lactating human breast and in breast cancer

**DOI:** 10.1038/sj.bjc.6600213

**Published:** 2002-04-08

**Authors:** C Zammit, R Coope, J J Gomm, S Shousha, C L Johnston, R C Coombes

**Affiliations:** Cancer Research (UK) Laboratories, Department of Cancer Medicine, Imperial College, Hammersmith Hospital, Du Cane Road, London W12 0NN, UK

**Keywords:** fibroblast growth factor 8, human breast cancer, lactation

## Abstract

Fibroblast growth factor 8 can transform NIH3T3 cells and its expression has been found to be associated with breast and prostate cancer. Following our finding that fibroblast growth factor 8 mRNA expression is increased in breast cancer, we have undertaken an immunohistochemistry study of fibroblast growth factor 8 expression in a series of human breast tissues and other normal tissues. Our findings confirm increased expression of fibroblast growth factor 8 in malignant breast tissue but also show significant fibroblast growth factor 8 expression in non-malignant breast epithelial cells. No significant difference in fibroblast growth factor 8 expression was found between different grades of ductal carcinoma, lobular carcinoma and ductal carcinoma *in-situ* or cancer of different oestrogen receptor, progesterone receptor or nodal status. The highest levels of fibroblast growth factor 8 expression were found in lactating breast tissues and fibroblast growth factor 8 was also detected in human milk. A survey of other normal tissues showed that fibroblast growth factor 8 is expressed in the proliferative cells of the dermis and epithelial cells in colon, ovary fallopian tube and uterus. Fibroblast growth factor 8 appears to be expressed in several organs in man and appears to have an importance in lactation.

*British Journal of Cancer* (2002) **86**, 1097–1103. DOI: 10.1038/sj/bjc/6600213 www.bjcancer.com

© 2002 Cancer Research UK

## 

The fibroblast growth factors (FGFs) form a family of at least 23 growth regulatory proteins that share 35–50% amino acid sequence identity. They induce proliferation and differentiation in a wide range of cells of epithelial, mesodermal and neuroectodermal origin (Basilico and Moscatelli 1992; Tanaka *et al*, 1992; Hu *et al*, 1998; Powers *et al*, 2000). FGF8 was originally isolated from the conditioned medium of an androgen dependent mouse mammary carcinoma line (SC-3) as an androgen induced growth factor (AIGF) and was later assigned as a member of the fibroblast growth factor family on the basis of structural similarity (Tanaka *et al*, 1992). FGF8 appears to have an important role in embryogenesis and is expressed in several areas of the developing mouse and may play a critical role in gastrulation and the development of the face, limb and central nervous system (Vogel *et al*, 1996; Neubuser *et al*, 1997; Meyers *et al*, 1998; Ye *et al*, 1998; Tucker *et al*, 1999; Sun *et al*, 1999). Little expression of FGF8 has been found in adult mouse tissues with comparatively low amounts detected only in the ovaries and testes (MacArthur *et al*, 1995a; Lorenzi *et al*, 1995).

We have been interested in assessing the role of fibroblast growth factors in the normal and malignant human breast. Previous work has shown that FGF1 and FGF2 are expressed in the normal breast, implying roles for these growth factors in maintaining the structure of the normal ducts (Gomm *et al*, 1991, 1997; Coope *et al*, 1997). In breast cancer, there is a decrease in the amount of FGF1 and FGF2 present in breast tissue (Bansal *et al*, 1995; Yiangou *et al*, 1997a). However, in the case of FGF1, there is evidence for some remaining in a more functionally available form in breast cancers (Smith *et al*, 1994; Coope *et al*, 1997). FGF7 is found in similar amount in both malignant and non-malignant breast tissues. It is produced by stromal fibroblasts and acts via the KGF receptor (FGFR2-IIIb) on epithelial cells (Bansal *et al*, 1997). Other studies show no expression of FGF3 and FGF4 in human breast cancers but expression of all other FGFs in at least a small proportion of breast cancers (Penault-Llorca *et al*, 1995). All four of the FGFRs are expressed to some degree in breast cell lines (McLeskey *et al*, 1994; Johnston *et al*, 1995). Although amplification of the *fgfr4* gene has been found in 10% of breast cancers, we have previously shown that equivalent levels of FGFR-4 are present in the epithelial cells of malignant and non-malignant breast sections (Jaakkola *et al*, 1993; Coope *et al*, 1997). In the case of FGFR-1, significant changes in the isoform of FGFR-1 expressed occur on malignant transformation although the level of expression appears to stay the same (Coope *et al*, 1997; Yiangou *et al*, 1997b).

One group of splice variants of the FGFR family involves the use of alternative exons encoding the carboxyl-terminal half of the third immunoglobulin domain. This form of splice variation occurs for FGFR-1, FGFR-2 and FGFR-3 so that IIIb and IIIc forms of these receptors have very different ligand binding properties (Miki *et al*, 1992; Werner *et al*, 1992; Chellaiah *et al*, 1994). These two isoforms appear to be expressed in a mutually exclusive fashion, with cells of mesenchymal origin expressing IIIc variants of FGFR2 and FGFR3 whereas epithelial cells express the IIIb isoform (Pekonen *et al*, 1993; Savagner *et al*, 1994). The interaction of several splice variants of FGF8 with high affinity receptors has been investigated and FGF8b has been shown to have the widest receptor binding properties, activating FGFR2-IIIc as well as FGFR3-IIIc and FGFR4 (MacArthur *et al*, 1995b).

FGF8 was identified as an oncogene on the basis of overexpression of FGF8 in NIH3T3 cells leading to focus formation, growth in soft agar and tumour formation in nude mice (Kouhara *et al*, 1994). FGF8 was subsequently found to act as a proto-oncogene co-operating with *Wnt 1* in mouse mammary tumorigenesis (Kapoun and Shackleford, 1997). Transgenic mice in which FGF8 was expressed under an MMTV promoter developed breast cancer over a period of months implying that FGF8 may be able to promote breast cancer in mice (Daphna-Iken *et al*, 1998).

Previous work has shown that FGF8 is expressed by prostate and breast cancer cell lines (Tanaka *et al*, 1995; Schmitt *et al*, 1996). Some investigators have demonstrated an involvement in malignant prostate disease since FGF8 was detected in prostatic cancers, but not in benign prostatic hypertrophy (Leung *et al*, 1996). Further studies indicated that FGF8 over-expression was an indicator of poor prognosis (Dorkin *et al*, 1999). However, other groups have failed to find this correlation between tumour grade and level of FGF8 expression (Tanaka *et al*, 1998; Wang *et al*, 1999). We have previously studied the expression of FGF8 mRNA in a series of malignant and non-malignant breast tissue and found that FGF8 transcripts are more frequently detected in malignant tumours and at a higher level than benign tissue (Marsh *et al*, 1999). In this study, we have extended our investigations into the role of FGF8 in the human breast by using antibodies against FGF8 to detect its presence in human breast samples. We find low levels of FGF8 in normal breast epithelial cells and the level of staining appears to be increased in some breast cancers. However, the highest levels of FGF8 expression were found in lactating mammary glands, indicating a possible role for this growth factor in lactation.

We have also investigated the expression of FGF8 in normal human tissues using immunohistochemistry in order to clarify whether FGF8 also acts as a normal regulator of cell activity. Our findings indicate that FGF8 is widely expressed in the normal adult and is likely to be important in the regulation of mature tissues as well as in their development.

## MATERIALS AND METHODS

### Tissues

Paraffin blocks of 124 human breast tissues were used in this study. The details of the 85 breast cancer patients are shown in Table 1

**Table 1:**
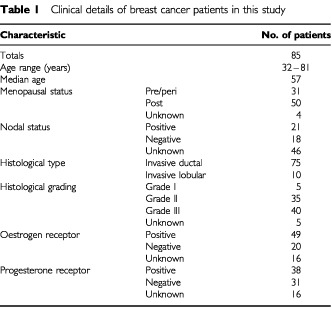
Clinical details of breast cancer patients in this study

. Eight cases of invasive ductal carcinoma occurred in lactating tissue. The remaining tissues were from five cases of fibroadenoma, 17 normal tissues from reduction mammoplasty tissue, 11 normal lactational tissues and six cases of DCIS. Of the 85 carcinoma tissues, 54 contained elements of adjacent normal tissue.

Paraffin sections of normal human tissues were also analysed and included skin, rectum, duodenum, ovary, cervix, endometrium and fallopian tube.

### Materials

Human milk was obtained from the milk bank at the post-natal department of Queen Charlotte's Hospital/London. FGF8 protein and anti-FGF8 monoclonal antibody were obtained from R&D systems (Abingdon, UK). All other chemicals were from Sigma (Poole, UK).

### Immunohistochemistry using a monoclonal antibody against FGF8

Paraffin sections were dewaxed in xylene and hydrated through graded alcohol and heated under pressure in 0.01 M citrate buffer pH 6.0 to reveal the antigen. Endogenous avidin and biotin were blocked using the avidin/biotin blocking kit (Vector). Non-specific binding was blocked with 10% horse serum and 5% BSA in PBS for 30 min. The sections were then treated for 16 h at 4°C with 1 μg ml^−1^ anti-FGF8 monoclonal antibody (R&D) in PBS. Control sections were incubated with 1 μg ml^−1^ non-immune mouse IgG (Sigma). Samples were washed three times in PBS and incubated with biotinylated anti-mouse IgG diluted in PBS with 10% human serum for 30 min at room temperature. After washing three times in PBS, the sections were incubated in ABC reagent (Vector Laboratories) for 1 h at room temperature. After washing three times in PBS, staining was visualised by addition of a 0.05% solution of 3,3′-diaminobenzidine (DAB) in PBS containing 001% hydrogen peroxide. Sections were then counterstained with Gill's haematoxylin, dehydrated, cleared and mounted.

Blocking experiments were performed by incubating the anti-FGF8 monoclonal antibody at 1 μg ml^−1^ with 100 μg ml^−1^ of recombinant FGF8 (R&D) for 16 h at 4°C. The antibody was then used to stain sections using the protocol described above.

### Scoring of staining

The level of staining was recorded as 0, +, ++ or +++ with 0 indicating no staining and +++ maximal staining. Scoring was checked by a histopathologist, experienced in breast disease who was blinded to the preliminary scoring results. Comparison of staining levels in oestrogen receptor positive and negative tumours or progesterone receptor positive and negative tumours was done using the Mann Whitney *U*-test. These receptor analysis were done routinely with primary tissue analysis at time of surgery by the histopathology laboratory at Charing Cross Hospital, London, using conventional techniques.

### Western blot detection of FGF8

Samples containing recombinant FGF or protein precipitated from milk were run on a 12% polyacrylamide gel and then transferred onto nitrocellulose for 16 h at 200 mA. The nitrocellulose was blocked by incubating with 3% casein in PBS with 0.1% Tween 20 for 1 h. The blot was probed with monoclonal anti-FGF8 (R&D) for 1 h and was washed three times in PBS. Incubation with an anti-mouse IgG-horseradish peroxidase conjugate (Sigma) was followed by extensive washes in PBS with 0.1% washes in PBS with 0.1% Tween 20 and development with ECL reagents (Amersham). Milk protein samples were prepared by centrifuging milk at 1000 **g** for 10 min to separate fat from milk. The semi-skimmed milk was filtered through a Microcon 100 to eliminate proteins of a molecular weight higher than 100 kDa. The milk solution was acidified to pH 4.6 using 1 M HCl to precipitate casein. This was centrifuged at 400 **g** for 30 min and the supernatant collected and neutralised to pH 7.6 with NaOH. The resulting proteins were used in Western analysis.

## RESULTS

### Specificity of the monoclonal antibody against FGF8

**Figure 1:**
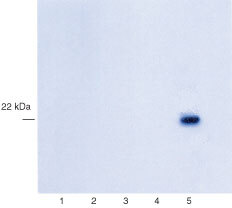
Western blots showing the specificity of the anti-FGF8 antibody. One hundred ng of FGF1 (lane 1), FGF2 (lane 2), FGF7 (lane 3), FGF9 (lane 4) and FGF8 (lane 5) were run on a 15% polyacrylamide gel and transferred onto nitrocellulose filters. These were probed with anti-FGF8 monoclonal antibody.

The specificity of the antibody to be used in immunohistochemistry was tested for its cross-reactivity against members of the FGF family by Western blotting. Equal amounts of recombinant FGF1, FGF2, FGF7, FGF8 and FGF9 were run on a 12% polyacrylamide gel. After transfer to nitrocellulose, the blot was probed using the monoclonal antibody against FGF8. No cross-reactivity with FGF 1, 2, 7 or 9 was seen (Figure 1

).

### Expression of FGF8 in human breast tissues

We have previously reported that FGF8 mRNA is expressed at a higher level in breast cancer cells than in normal breast epithelial cells (Marsh *et al*, 1999). Following these observations, we have used a monoclonal antibody against FGF8 in immunohistochemistry to assess the expression and distribution of FGF8 in a series of malignant and non-malignant human breast tissues. Details of the breast cancer patients studied are shown in Table 1 and in addition, 17 normal reduction mammoplasty tissues, five cases of fibroadenoma and 11 normal lactating breast tissues were analysed.

**Figure 2:**
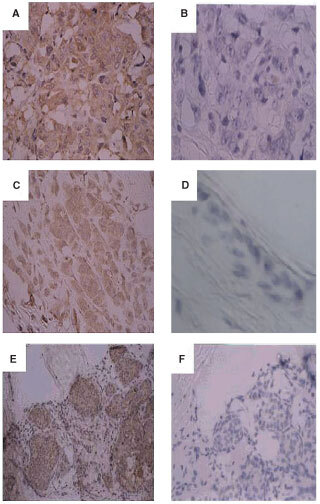
Expression of FGF8 in human breast cancer tissue. Paraffin sections of invasive ductal carcinoma (**A**,**B**), invasive lobular carcinoma (**C**,**D**) and ductal carcinoma *in situ* (DCIS) (**E**,**F**), were analysed by immunohistochemistry using an antibody against FGF-8 (**A**,**C**,**E**) or the equivalent concentration of non-immune mouse IgG (**B**,**F**). (**D**) Shows absence of anti-FGF8 staining after pre-incubation with 100-fold excess of recombinant FGF8. (Original magnification **A**–**D**, ×400; **E**, **F**, ×200).

Immunostaining of human breast sections from invasive ductal carcinoma (Figure 2A,B

), invasive lobular carcinoma (Figure 2C,D) and DCIS (Figure 2E,F) showed expression of FGF8 in epithelial cells but not of adjacent stromal elements (Figure 2A,C,E). FGF8 was not found in the nuclei of cells and gave a diffuse cytoplasmic staining pattern. The staining appeared to be specific since pre-incubation of anti-FGF8 with excess recombinant FGF8, blocked the staining (Figure 2D) and non-immune mouse IgG at the same concentration gave negative staining (Figure 2B,F).

**Figure 3:**
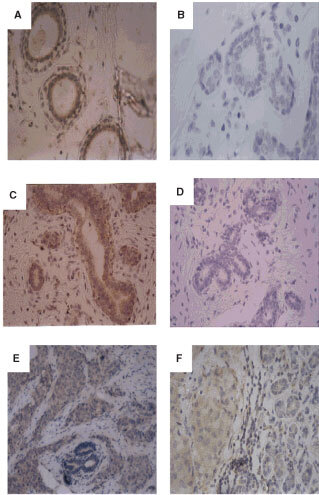
Expression of FGF8 in non-malignant human breast tissue. Paraffin sections of normal breast tissue from reduction mammoplasty tissue (**A**,**B**), fibroadenoma (**C**,**D**) and paraffin sections containing elements of both invasive ductal carcinoma and adjacent normal breast tissues (**E**,**F**), were analysed by immunohistochemistry using an antibody against FGF8 (**A**,**C**,**E**,**F**) or the equivalent concentration of non-immune mouse IgG (**B**,**D**). (Original magnification **A**–**B**, ×400; **C**–**F** ×200).

FGF8 immunostaining could also be detected in non-malignant breast tissues. In cases of reduction mammoplasty tissue, cytoplasmic FGF8 staining was seen in epithelial cells but not in myoepithelial cells or in stromal elements (Figure 3A

). Fibroadenoma tissues also showed cytoplasmic FGF8 immunostaining in the epithelial cells (Figure 3C). In sections where normal elements of breast tissues could be seen adjacent to invasive carcinoma, higher levels of FGF8 staining were consistently seen in carcinoma elements compared to normal elements (Figure 3E,F). The intensity of FGF8 immuno-reactivity was graded as 0, +, ++ or +++ and a score was given to the tissues analysed.

**Figure 4:**
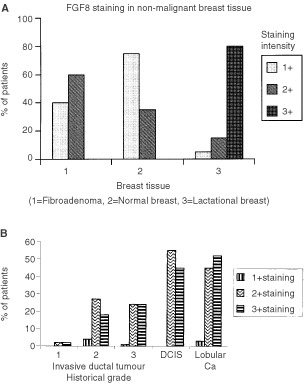
Histograms showing the level of FGF8 immunoreactivity in malignant and non-malignant breast tissue. Tissues were scored as +, ++ or +++ for FGF8 staining. (**A**) Shows percentages of patients with each level of staining in: 1=Fibroadenoma; 2=Normal breast tissue (reducton mammoplasty); 3=Lactating breast tissue. (**B**) Shows FGF8 staining patterns in Invasive Ductal Carcinoma Grade 1, 2 and 3; DCIS and Lobular carcinoma.

A comparison of the level of FGF8 immuno-reactivity in fibroadenoma, normal breast tissue from reduction mammoplasty and lactational breast tissue (Figure 4A

); DCIS, lobular breast cancer tissue and different grades of invasive ductal carcinoma is shown (Figure 4B). In general, we found an increase in FGF8 expression in malignant breast tissues compared to non-malignant. Hence comparatively low levels of FGF immunoreactivity were found in normal breast tissue from reduction mammoplasty tissue with the majority of tissues registering as + and ++. Fibroadenoma tissues also showed similar low levels of FGF8 expression as normal breast tissue. DCIS, lobular carcinoma and ductal carcinoma tissues more commonly registered as ++ and +++. We observed no significant difference in FGF8 expression between invasive lobular and invasive ductal carcinoma. No significant difference in the level of FGF8 immunoreactivity was seen in invasive ductal carcinomas of histological grades II and III. The Grade I breast carcinoma specimens showed similar low expression of FGF8 as normal breast tissues but statistical comparative analysis with higher tumour grades was not possible as numbers were small. No significant difference in FGF8 expression was seen in cancers with different oestrogen receptor status (*P*=0.5), progesterone receptor status (*P*=0.0534) or nodal status (*P*=0.138).

### FGF8 expression in lactating breast

**Figure 5:**
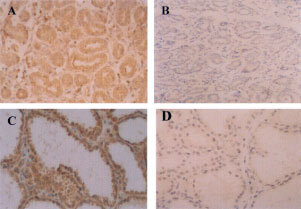
FGF8 staining in lactating mammary gland. Paraffin sections of lactating mammary gland tissue were stained with an antibody against FGF8 (**A**,**C**) or with the equivalent concentration of non-immune mouse IgG (**B**,**D**). (Original magnification **A**, **B**, ×200; **C**, **D**, ×400).

**Figure 6:**
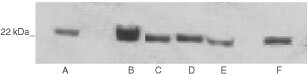
Western blots demonstrating the presence of FGF8 in human breast milk. Proteins from human milk samples were run on a 12% polyacrylamide gel, transferred onto nitrocellulose and probed using the monoclonal antibody against FGF8. Lanes **A**–**E** contain milk proteins and lane F contains 100 ng of recombinant FGF8.

A series of sections from lactating breasts were stained for FGF8 and all showed levels of FGF8 expression higher than any of the normal or malignant breast sections. Again cytoplasmic staining of epithelial cells was seen with myoepithelial cells and stromal components being negative (Figure 5

). Protein was isolated from samples of human milk and Western blot analysis was used to assess whether FGF8 was present. Five samples of milk analysed all contained FGF8 (Figure 6

).

### FGF8 expression in other tissues

**Figure 7:**
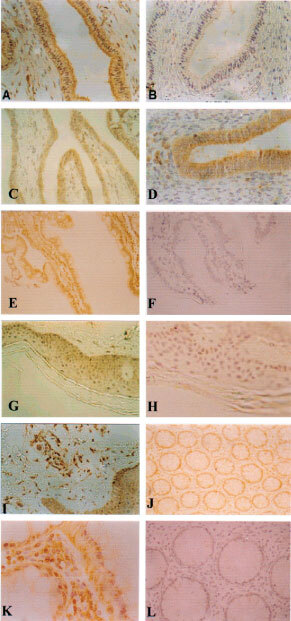
Immunostaining of different normal human tissues. Shown are section of endometrium (**A**,**B**); fallopian tube (**C**); endocervix (**D**); duodenum (**E**,**F**); skin (**G**,**H**); skin with sweat glands (**I**); rectum (**J**,**K** and **L**). (Original magnification **A**–**H**, **J**,**L**, ×200; **I**,**K**, ×200.

Previous reports concerning FGF8 expression in adult mouse tissues showed only a small amount of FGF8 expression in ovaries and testes (Lorenzi *et al*, 1995). To see whether this is a true reflection of FGF8 expression in normal human tissues, an immunohistochemical study was carried out. FGF8 immunoreactivity was present within the epidermal layer of the skin (Figure 7G,I

). The greatest levels of FGF8 staining was found towards the bottom of the epidermal layer containing dividing cells rather than in the more differentiated upper layers. Sections of skin containing sweat glands showed high FGF8 staining of the sweat glands (Figure 7I).

FGF8 expression was also detected within the human bowel. Sections of rectum showed immunoreactivity at the base of goblet cells and in surface epithelial cells (Figure 7J,K). Interstitial lymphocytes and plasma cells were stained positive. In sections of duodenum, columnar absorptive cells showed positive cytoplasmic staining mainly at the base but also in more superficial parts of some cells (Figure 7E). Intestinal Goblet cells and Paneth cells showed less positive staining, mainly at the base of the cells.

In the ecto-cervix, the stratified squamous layer showed faint staining but the keratin layer was clear. More immunoreactivity was found in the endocervix with columnar cells lining glands showing positive staining (Figure 7D). FGF8 expression was also seen in the columnar epithelial cells of the fallopian tube (Figure 7C). Within the ovary, high levels of FGF8 were seen in the Corpus Luteum. In the endometrium, glands showed positive staining (Figure 7A). The endometrial stroma was negative, however the myometrium showed positive staining.

## DISCUSSION

FGF8 is of potential interest in breast biology since it was first isolated from a mouse mammary carcinoma cell line and its expression is regulated by steroid hormones; in particular dihydroxytestosterone (Tanaka *et al*, 1992; Payson *et al*, 1996). FGF8 is able to function as an oncogene, causing transformation of NIH3T3 cells and inducing breast tumours in transgenic mice expressing FGF8 under an MMTV promoter (Kouhara *et al*, 1994; Daphna-Iken *et al*, 1998). FGF8 expression appears to be associated with human carcinoma of the prostate since higher levels of FGF8 were detected in malignant prostate disease (Leung *et al*, 1996; Tanaka *et al*, 1998). However, another group found FGF8 expressed in benign prostatic hypertrophy as well as prostate cancer (Wang *et al*, 1999). FGF8 expression has also been shown in a proportion of breast cancers and normal breast tissues (Tanaka *et al*, 1998; Marsh *et al*, 1999). Although immunohistochemical studies have been carried out showing a correlation between the grade of prostate cancers and the amount of FGF8 expression, such extensive studies have not previously been carried out using human breast tissue. Our studies show that although FGF8 expression appears to be slightly higher in malignant breast disease than in normal breast tissue, FGF8 is expressed in normal epithelial cells and in fact the highest level of FGF8 expression were found in lactating mammary gland.

The results of the staining are consistent with previously published reports. mRNA encoding FGF8 mRNA has been found expressed in the epithelial cells of breast cancer using *in situ* hybridisation and we find a similar expression pattern in the epithelial component of normal and malignant breast tissue (Marsh *et al*, 1999). It has been reported that FGF8 mRNA is present in a proportion of both malignant and non-malignant breast tissues (Wu *et al*, 1997; Tanaka *et al*, 1998). Our findings show that the majority of breast epithelial cells contain considerable levels of FGF8 protein.

The staining pattern observed indicates that FGF8 has a cytoplasmic or membrane bound localisation but is not seen in the nucleus of epithelial cells. This is in contrast with several other members of the FGF family. FGF 1, 2 and 3 have been detected in the cell nucleus (Cao *et al*, 1993; Presta *et al*, 1993; Antoine *et al*, 1997). Eight different splice variants of FGF8 are possible in the mouse but in man due to a stop codon only four of these forms are possible (Crossley and Martin, 1995; Gemel *et al*, 1996). Previous studies have indicated that FGF8b is the predominant form in breast tissue (Marsh *et al*, 1999) and that FGF8b has been shown to have the greatest transforming ability (MacArthur *et al*, 1995b). The monoclonal anti-FGF8 antibody used in this study (R&D Systems) was produced from a mouse hybridoma in which the immunogen was an *E. coli*-derived recombinant mouse fibroblast growth factor 8b.

Transgenic mice overexpressing FGF8 under an MMTV promoter have been reported to produce lobular carcinoma of the breast (Daphna-Iken *et al*, 1998). We therefore compared the levels of FGF8 staining in groups of invasive ductal and invasive lobular carcinoma. We failed to detect any difference in FGF8 expression in the two groups, suggesting that the causal relationship implied in mice may not be important in humans. A comparison of FGF8 expression in different histological grades of ductal carcinoma also showed no difference between staining in different grades of breast tumour. This is in contrast to a prostate cancer report in which higher levels of FGF8 were found in less differentiated cancers (Leung *et al*, 1996; Tanaka *et al*, 1998; Dorkin *et al*, 1999). It is known that FGF8 expression is regulated by androgens (Tanaka *et al*, 1992). We see no evidence of higher FGF8 expression correlating with loss of oestrogen or progesterone receptors. Previous reports have shown that dihydoxytestesterone but neither oestrogen nor progesterone was able to upregulate FGF8 expression (Payson *et al*, 1996). In view of these findings, it is not surprising that FGF8 expression fails to correlate with ER or PR status.

The highest levels of FGF8 expression were detected in lactating mammary gland, strongly implicating FGF8 as having an important role in lactation. A large number of hormones and related peptides are present in milk and colostrum; insulin, epidermal growth factor (EGF), insulin-like growth factor I (IGF-I) and cortisol are abundantly present (Xu, 1996). FGF8 has been implicated in embryological development especially gastrulation suggesting roles in brain, limb and facial development (Ohuchi *et al*, 2000). FGF8 has also been implicated in morphogenic outgrowth of the hepatic endoderm (Jung *et al*, 1999). However in the early post natal period the gastrointestinal tract is still in a process of development and maturation which seem to be under the influence of milk ingested (Xu, 1996). With the presence of high quantities of FGF8 in human milk, FGF8 along with other growth/hormonal factors may be implicated in this process.

We also used immunohistochemistry to determine the expression of FGF8 in normal adult tissues. By this method, we were able to detect FGF8 expression in the epithelial cells of many of the tissues examined. The widespread expression pattern suggests that FGF8 has physiological roles in adult tissue as well as in development.

Previous studies have used Northern blotting and *in situ* hybridisation to detect FGF8 expression. High levels of FGF8 mRNA were found during development in the mouse whereas in the adult mouse a little expression was found in ovary and testis (Lorenzi *et al*, 1995). Our results confirm expression of FGF8 in the ovary, however more widespread expression was apparent. This could indicate that the immunohistochemical method used is more sensitive or it could reflect a difference between the two species. Our finding of FGF8 expression in normal breast tissue is in agreement with previous reports studying mRNA and protein levels (Tanaka *et al*, 1998; Marsh *et al*, 1999). Since we have not included foetal tissue in this study, it is possible that higher levels are present in development.

Other members of the FGF family are expressed in adult tissues and in many cases the activity of such growth factors is tightly regulated to prevent inappropriate cell responses. In the case of FGF1 and FGF2, release is the point of regulation since these growth factors lack a signal peptide and are therefore not secreted by the classical pathway. Release of these growth factors is possible but appears to be regulated by binding of integrins to extracellular matrix components and other factors such as oestrogen stimulation (Mousa *et al*, 1999).

Since FGF8 contains a signal peptide, its regulation is more likely to follow the model of FGF7. FGF7 binds to only one FGF receptors, the IIIb isoform of FGFR-2. Since ephithelial cells exclusively express FGFR2-IIIb and FGF7 is secreted by fibroblasts, FGF7 is likely to act in a paracrine fashion (Bansal *et al*, 1997). A similar situation may occur in the case of FGF8, which is expressed principally in epithelial cells with very little expression seen in stromal tissues in this study and in other reports (Tanaka *et al*, 1998; Marsh *et al*, 1999). Its receptor binding properties have been reported previously and the highest affinity receptors are FGFR2-IIIc, FGFR3-IIIc and FGFR4 (Ornitz *et al*, 1996). Both FGFR2-IIIC and FGFR3-IIIc receptors are expressed predominantly in fibroblasts (Yan *et al*, 1993; Scotet and Houssaint, 1995). Again this raises the likelihood of FGF8 operating through a paracrine interaction, being released by epithelial cells and acting on surrounding fibroblasts. FGFR4 is another receptor for FGF8.We have previously shown that FGFR4 is present in epithelial cells of normal (including lactating breast) and malignant breast tissue (Coope *et al*, 1997), therefore an autocrine loop of FGF8/FGFR4 is also possible.

Detailed immunohistochemical staining for FGFRs has been carried out on a range of normal human tissues and show widespread expression of this family of proteins in the normal adult (Hughes, 1997). Within the skin, high levels of FGFR2 were found in dermal fibroblasts. FGF8 expression is found in the basal level of the skin, providing a potential paracrine interaction for FGF8. A high level of FGFR3 expression was found in the cervix including its presence in stromal fibroblasts. The presence of FGF8 in the epithelial cells of the cervix suggests a similar paracrine interaction with the stroma. In the duodenum, expression of FGFR2 and FGFR3 was found in the muscularis mucosae giving potential interactions for the FGF8 released by columnar epithelial cells. High level of FGFR-3 were also detected in the colon. No expression of FGFR-2 or FGFR-3 was found in the ovary.

The widespread expression of FGF8 in normal tissues implicates this growth factor as having a physiological role in the adult as well as during development. Because of its production by epithelial cells and the presence of its receptors on fibroblasts, it probably has a paracrine mode of action in skin, cervix, duodenum and rectum. Its function in the endometrium and ovary is less clear since FGF8 appears to be localised towards the apical surface of epithelial cells in these tissues.
